# Reclamation of hexavalent chromium using catalytic activity of highly recyclable biogenic Pd(0) nanoparticles

**DOI:** 10.1038/s41598-020-57548-z

**Published:** 2020-01-20

**Authors:** R. M. Tripathi, Sang J. Chung

**Affiliations:** 1 School of Pharmacy, Sungkyunkwan University, 2066 Seoburo, Jangan-gu, Suwon, Gyeonggido 16419 Republic of Korea; 2 Amity Institute of Nanotechnology, Amity University Uttar Pradesh, Sector 125, Noida, 201303 India

**Keywords:** Environmental sciences, Chemistry, Materials science, Nanoscience and technology

## Abstract

Hexavalent chromium is extremely toxic and increasingly prevalent owing to industrialisation, thereby posing serious human health and environmental risks. Therefore, new approaches for detoxifying high concentrations of Cr (VI) using an ultralow amount of catalyst with high recyclability are increasingly being considered. The catalytic conversion of Cr (VI) into Cr (III) was previously reported; however, it required a large amount of catalyst to reduce a low concentration of Cr (VI); further, pH adjustment and catalyst separation had to be performed, causing issues with large-scale remediation. In this study, an unprecedented eco-friendly and cost-effective method was developed for the synthesis of Pd nanoparticles (PdNPs) with a significantly narrow size distribution of 3–25 nm. PdNPs demonstrated the presence of elemental Pd with the zero oxidation state when analysed by energy-dispersive X-ray analysis and X-ray photoelectron spectroscopy. The PdNPs could detoxify a high concentration of Cr (VI), without the need to adjust the pH or purify the nanoparticles for reusability. The reusability of the PdNPs for the catalytic conversion of Cr (VI) into Cr (III) was >90% for subsequent cycles without the further addition of formic acid. Thus, the study provides new insights into the catalytic reclamation of Cr (VI) for industrial wastewater treatment.

## Introduction

Heavy-metal pollution is a serious hazard and a global issue due to the increase in industrial and agricultural activities. Several toxic pollutants, such as inorganic ions, metals, and synthetic organic matter, are released into water. Among these, Cr (VI) is the most dangerous toxic pollutant. Cr is used for various purposes, such as leather tanning, paint formulation, wood preservatives, steel fabrication, and metal finishing, resulting in considerable Cr-based water contamination. The oxidation state of Cr significantly affects its toxicity. Of the two primary oxidation states, i.e., Cr (III) and Cr (VI), Cr (VI) is more toxic and carcinogenic to humans and animals. A significantly low concentration of Cr (III) is required by humans, as it plays an important role in glucose metabolism; however, a high concentration of Cr (III) is toxic^1^. The highest acceptable concentration of Cr in drinking water is 50 parts per billion, as recommended by the World Health Organization^2^. The remediation of Cr (VI) is a critical research challenge. A physicochemical method was developed for the adsorption of Cr; however, it is expensive; further, Cr (VI) is simply transferred and not removed^3^. The bioremediation of Cr (VI) using bacteria is viable and cost-effective; however, the resulting waste contains bactericidal toxicants, which limits the efficiency and applicability of this method^4^.

Currently, scientists are working towards the catalytic reduction of Cr (VI) to Cr (III). Compared to Cr (VI), Cr (III) has lower toxicity and mobility; moreover, a minute amount of Cr (III) is required for sugar and lipid metabolism in humans and other animals^5,6^. The nanomaterial-based catalytic reduction of Cr (VI) has attracted considerable attention because of its advantages over physicochemical- and bioremediation-based methods. Pd nanoparticles (PdNPs) have been extensively applied on various support materials, such as MIL-101^7^, alpha-Al_2_O_3_ films^5^, polymer nanofibers^8^, and surface-functionalised SiO_2_^9^. The effectiveness of PdNPs in the reduction of Cr (VI) (in the presence of formic acid) is owing to their distinguished features of high selectivity and activity in catalytic hydrogenation reactions^9–11^. The reduction of Cr (VI) by PdNPs in the presence of formic acid involves two steps: the catalytic hydrogenation of formic acid (HCOOH → H_2_ + CO_2_)^12–16^ and the reduction of Cr (VI) to Cr (III) through a H_2_ transfer pathway on the surface of PdNPs (Cr_2_O_7_^2−^ + 8H^+^  + 3H_2_ → 2Cr^3+^  + 7H_2_O)^5,17,18^. PdNPs were decorated on graphene oxide and exhibited remarkable reusability (>90% at fifth reuse)^19^.

Generally, reusability is challenging owing to the need for recovering, purifying, and drying the catalyst. Additionally, chemical methods for synthesising nanocatalysts/photocatalysts cause environmental pollution, as hazardous chemicals are required for the synthesis process. Therefore, researchers focused on nontoxic, cost-effective, facile, and eco-friendly methods for the synthesis of nanomaterials. Typically, bacteria, fungi, and plant extracts are used to synthesise various types of nanomaterials^20–24^. Plant extracts have gained significant attention compared to bacteria and fungi because they do not require culture maintenance.

The objectives of this study were to develop a simple, cost-effective, and eco-friendly method for the biosynthesis of PdNPs using a leaf extract of *Erigeron canadensis L* (*E. canadensis*) and to investigate their effectiveness and reusability for the reduction of Cr (VI) without the separation of PdNPs. The novelty of the present work is indicated by Table 1. *Erigeron species* is a source of γ-pyranone derivatives, flavonoids, and phenolic acids^25^, and is important for the synthesis of nanoparticles^26–28^. This plant has medicinal value in the treatment of indigestion, hematuria, enteritis, and epidemic hepatitis^29^. A flower extract of *E. annuus* (L.) Pers was used as a reducing and capping agent for the synthesis of silver and gold nanoparticles^30^. Previous studies considered the separation of catalysts/photocatalysts, which resulted in a difficult, expensive, and time-consuming reusability process. The proposed method does not require recovery, purification, or drying of biogenic PdNPs. Additionally, it can be applied to industrial wastewater treatment because once the biogenic PdNPs are added to the wastewater, additional PdNPs need not be added for several consecutive cycles, and no further addition of formic acid is required.

**Table 1:** Reduction of Cr (VI) by different catalytic nanomaterials.

Nanomaterial	Quantity of Cr (VI)	Quantity of nanomaterial	pH	Rate constant (min^−1^)	Reference
TiO_2_ nanocrystals (photocatalytic)	50 ppm	500 ppm	Either 2.7 or 7.0	—	^31^
Iron micro/nanostructure	100 ppm	1,500 ppm	2	0.286	^32^
Cobalt phosphate-sensitised inverse opal TiO_2_ (photocatalytic)	10 ppm	10 ppm	3	—	^33^
Magnetic mesoporous carbon-doped PdNPs	50 ppm	800 ppm	2	0.017	^34^
Cobalt nanoparticles supported on graphene	100 ppm	100 ppm	2	0.474	^35^
Sulfur nanoparticles	200 ppm	10 ppm	1–2	0.027	^36^
NiO nanostructure (photocatalytic)	130 ppm	400 ppm	7	0.0026	^37^
Nanoscale zerovalent iron supported on mesoporous silica	6 ppm	180 ppm	3 & 5	0.017	^38^
PdNPs	~147 ppm	0.5 ppm	3	—	^39^
Biogenic PdNPs	250 ppm	0.043 ppm	Not required	0.0971	Present work

## Results

### Ultraviolet–visible light spectroscopy analysis

**Figure 1:**
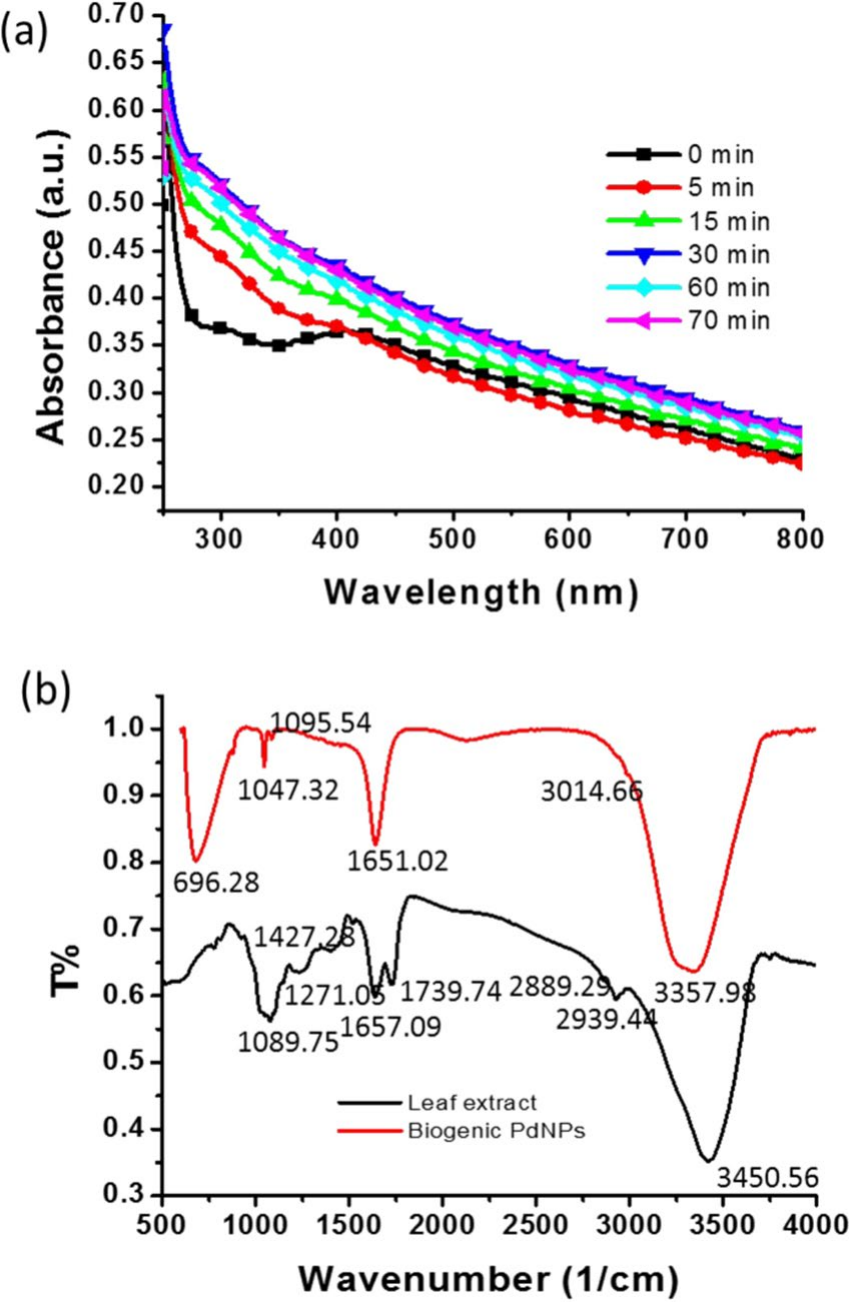
(**a**) Ultraviolet–visible (UV–vis) spectra for the biosynthesis of the Pd nanoparticles (PdNPs) as a function of time; (**b**) Fourier transform infrared spectra of the leaf extract of *Erigeron canadensis* and the biologically synthesised PdNPs.

The synthesis of the PdNPs was monitored at 0, 5, 15, 30, and 70 min by scanning the sample by ultraviolet–visible light (UV–vis) spectroscopy. The spectra revealed that 30 min was sufficient for synthesising the PdNPs. Figure 1a shows a distinct peak around 400 nm at 0 min, indicating the presence of Pd^2+^ ions in the solution; however, after 30 min, this peak disappeared. The sample was scanned after 70 min; however, no change was observed in the absorbance (Fig. 1a).

### Fourier transform infrared spectroscopy

The synthesised nanoparticles were scanned by Fourier transform infrared (FTIR) spectroscopy in the range of 500–4,000 cm^−1^ (Fig. 1b). The FTIR spectrum of the leaf extract exhibited a broad, intense peak at 3,450.56 cm^−1^, whereas in the spectrum of the PdNPs, this peak shifted to 3,357.98 cm^−1^, indicating –OH stretching^40^. The peak at 2,939.44 cm^−1^ in the leaf-extract spectrum corresponds to the C-H stretching of CH_2_ and CH_3_^41^. However, in the spectrum of the PdNPs, no peak was observed at 2,939.44 cm^−1^, suggesting the involvement of C-H stretching vibration in the formation of the PdNPs. A peak was observed at 1,739.74 cm^−1^, corresponding to C = O stretching of the aldehyde group. The band at 1,654.88 cm^−1^, in the case of the leaf extract, was shifted to 1,651.02 cm^−1^ in the spectrum of the PdNPs, corresponding to the stretching vibration of COO^−^. The leaf-extract spectrum exhibited a peak at 1,427.28 cm^−1^, corresponding to the N-H stretching vibration in the amide linkages of the protein; this peak was not observed for the PdNPs. The band at 1,271.05 cm^−1^ for the leaf extract was similar to that at 1,240 cm^−1^, which corresponds to the C-N stretching of amines^42^. This band was not observed for the PdNPs. The spectra of the PdNPs and leaf extract exhibited peaks at 1,095.54 and 1,089.75 cm^−1^, respectively, indicating a marginal shift. These peaks were similar to that at 1,074 cm^−1^ and indicate the presence of flavanones adsorbed on the surface of the nanoparticles^43^.

### Transmission electron microscopy

**Figure 2:**
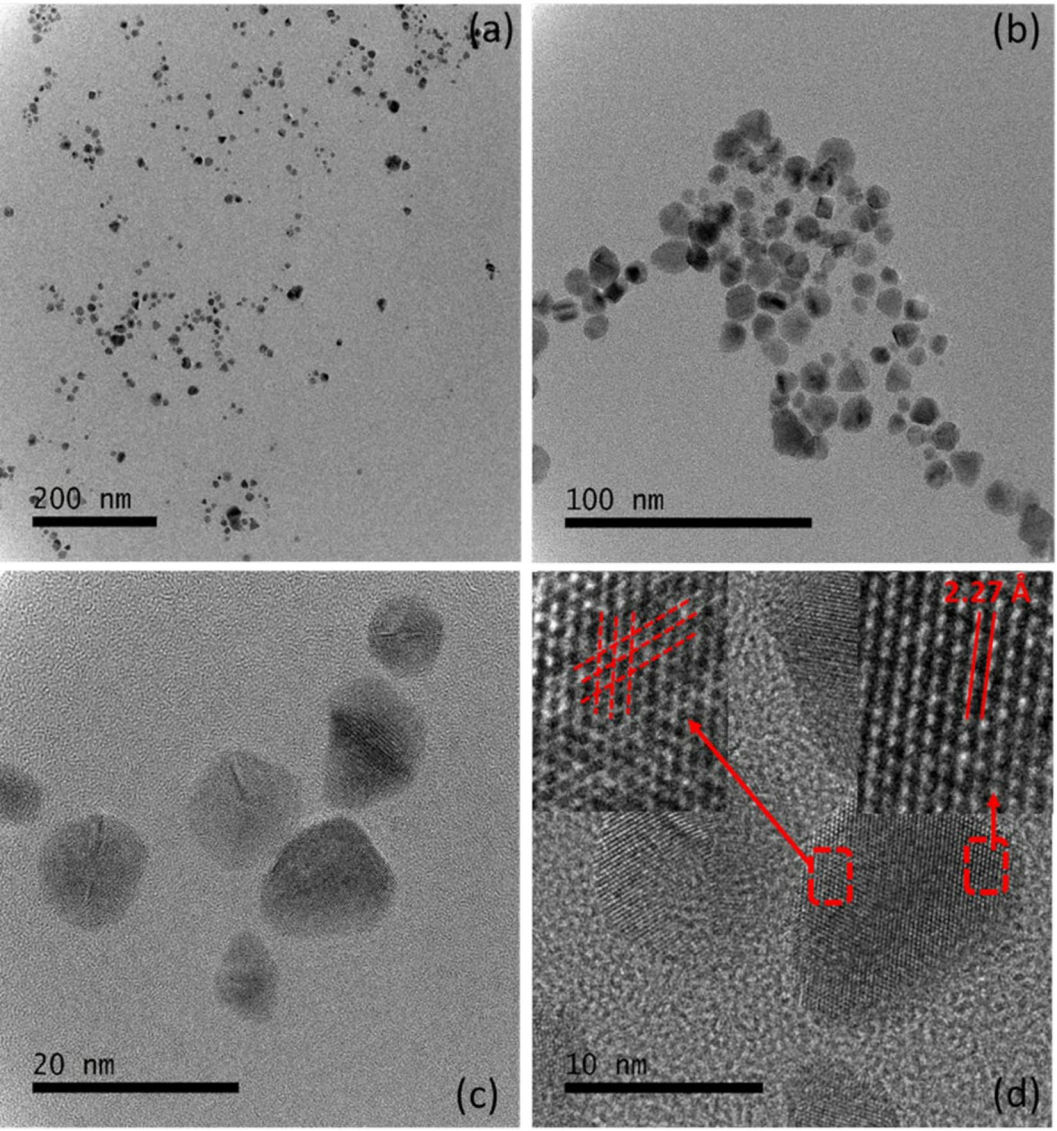
Transmission electron microscopy (TEM) images of the PdNPs at different magnifications: (**a**) Wide scan of the sample at 200 nm for a large view; (**b**) Scan focused on a small area of the sample; (**c**) High-resolution TEM (HR-TEM) image showing the crystalline nature of PdNPs; (**d**) HR-TEM image showing the d-spacing and polycrystalline nature of the biogenic PdNPs.

**Figure 3:**
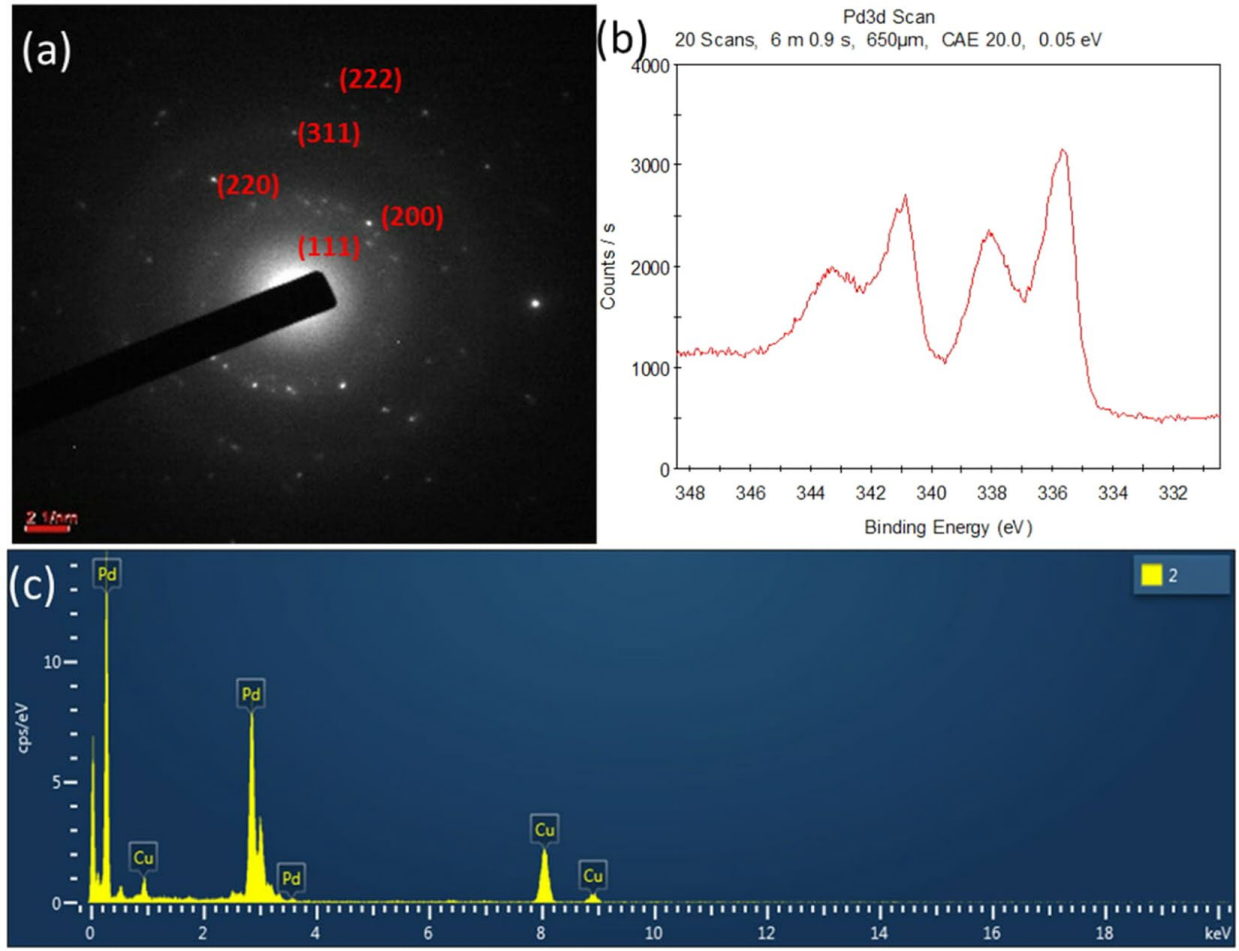
Biosynthesised PdNPs: (**a**) Selected-area electron diffraction pattern; (**b**) X-ray photoelectron spectra; (**c**) Energy-dispersive X-ray spectra.

A sample was prepared on a carbon-coated copper grid via drop-coating, and transmission electron microscopy (TEM) was performed for analysis of the size, morphology, and crystalline nature of the biosynthesised PdNPs. TEM images were obtained at various magnifications, which revealed the morphology of the nanoparticles (Fig. 2). The particles had a significantly narrow size distribution of 3–25 nm with an average size of 5 nm (Fig. 2a). High-magnification observations revealed that the nanoparticles had hexagonal, triangular, and spherical morphologies (Fig. 2b). In the high-resolution TEM (HR-TEM) analysis, all the particles exhibited the lattice-fringe characteristic of crystalline materials (Fig. 2c). The inset on the left of Fig. 2d shows cross lattice fringes, clearly indicating the polycrystalline nature of the nanoparticles. The inter-atomic spacing (d-spacing) of the biogenic PdNPs was determined to be 2.27 Å (Fig. 2d, inset on the right). These results indicate the unprecedented quality of the developed method for the synthesis of PdNPs. The selected-area electron diffraction pattern of the biosynthesised PdNPs indicates the crystalline nature of the nanoparticles and shows rings corresponding to the (111), (200), (220), (311), and (222) planes of Pd with a face-centred cubic structure (Fig. 3a).

### Energy-dispersive X-ray analysis and X-ray photoelectron spectroscopy

The elemental composition of the nanoparticles was analysed through energy-dispersive X-ray (EDX) analysis using a transmission electron microscope. The imaging capability of the microscope enabled selection of the specimen of interest. The EDX results were in the form of a graph of the X-ray counts (cps/eV) relative to the energy (keV). The EDX spectrum showed a strong signal of elemental Pd, indicating the purity of the biosynthesised PdNPs (Fig. 3c). There was another elemental signal corresponding to copper, owing to the copper grid used for the sample preparation. No other elemental signal was observed in the EDX spectrum, confirming the elemental purity of the biologically synthesised PdNPs. Supplementary Table S1 lists the elements detected through EDX, along with their compositions, elemental percentages, series, and k factors. X-ray photoelectron spectroscopy (XPS) was used to analyse the oxidation of the state of the biosynthesised PdNPs. The colloidal solution of nanoparticles was dried on a glass plate to prepare the sample for XPS analysis. The XPS profiles exhibited a peak at a binding energy of 335.6 eV, which is the characteristic binding energy of Pd(0) (Fig. 3b). This indicates that zero-valent PdNPs were synthesised.

### Potential biosynthesis mechanism

The potential biosynthesis mechanism of PdNPs is based on the presence of polyphenolic compounds in the leaf extract. Polyphenolic compounds exist in abundance in all parts of the plants, and play an important role in neutralising the effect of the reactive oxygen species^27,28^. The aqueous extract of the *E. canadensis* leaf has flavonoids and tannins, which show high antioxidative properties. As soon as the leaf extract was added into the palladium chloride solution, the reduction of Pd^2+^ to Pd^0^ by polyphenolic compounds occurred. The neutralised Pd ions experienced the physical phenomena of nucleation, which produced nanoparticles. FTIR illustrated a peak at 1,743 cm^−1^, corresponding to the C = O stretching of the aldehyde group. A band at 1,654.88 cm^−1^, in the case of the leaf extract, was shifted to 1,651.02 cm^−1^ in the spectrum of the PdNPs, corresponding to the stretching vibration of COO^−^. Figure 1b shows the comparative FTIR spectra of the leaf extract and synthesised nanoparticles, which evidently demonstrate the contribution of biomolecules. The FTIR analysis clearly reveals the participation of biological molecules for the synthesis of nanoparticles. However, additional experimental analysis would be required to understand the detailed biosynthesis mechanism.

### Catalytic reduction of Cr (VI)

The reduction of Cr (VI) with the combination of 0.26 M formic acid and 0.043 parts per million (ppm) PdNPs was evaluated. All the experiments were performed under ambient conditions, without adjusting the pH of the Cr (VI)-contaminated water. The catalytic reduction efficiency was analysed using the following formula.

1 $${\rm{Reduction}}\,{\rm{efficiency}}\,( \% )=1-({\rm{C}}/{{\rm{C}}}_{0})\times 100$$

**Figure 4:**
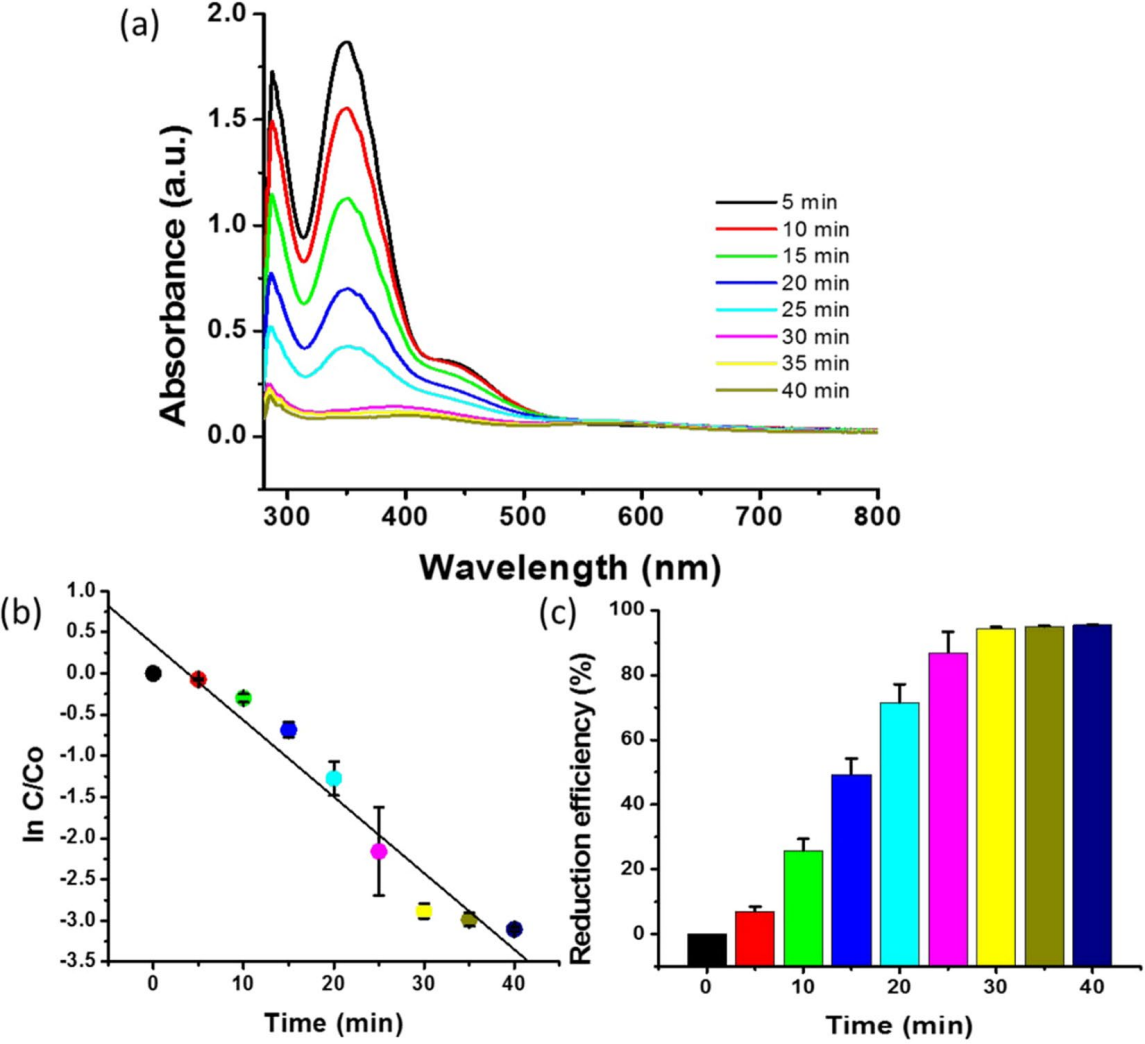
Catalytic activity of biogenic PdNPs: (**a**) Absorbance spectra changes at 350 nm for Cr (VI) in the presence of PdNPs as a function of time; (**b**) First-order linear plot of ln(C/C_0_) versus time; (**c**) Reduction efficiency (%) versus time.

The absorbance was measured as a function of time to quantitatively investigate the reaction kinetics of the catalytic reduction of Cr (VI) (Fig. 4a). Experiments were performed to analyse the catalytic activity of the biogenic PdNPs and to obtain error bars for the standard deviation (Fig. 4b,c). As soon as the PdNPs were added to the aqueous solution of Cr (VI) in the presence of formic acid, the yellow colour of Cr (VI) began to disappear and the absorption at 350 nm decreased drastically, clearly indicating the reduction of the Cr (VI) by the as-synthesised nanoparticles (Fig. 4a). The reduction efficiency was 95.53% after 40 min of incubation time (Fig. 4c). We analysed the catalytic activity up to 80 min; however, after 40 min, minimal reduction was observed (Supplementary Fig. S1). This reveals that 40 min of incubation was sufficient to reduce 250 ppm of Cr (VI). Therefore, all the experiments related to Cr (VI) reduction were performed for 40 min for analysing the catalytic activity of the PdNPs.

Figure 4b shows the experimental data fitted for the first-order kinetics. A quantitative analysis of the reaction kinetics of the catalytic reduction of Cr (VI) was performed by calculating ln(C/C_0_) at a specific time.

2 $$\mathrm{ln}({\rm{C}}/{{\rm{C}}}_{0})=-\,{\rm{kt}}$$

Here, k is the first-order rate constant, t is the visible-light illumination, and C_0_ and C are the concentrations of Cr (VI) in the reaction solution at times zero and t, respectively.

The rate constant (k) was analysed using the linear plot of ln(C/C_0_) versus the reduction time and estimated to be 0.0971 min^−1^ (Fig. 4b). Previous reports showed that a 88.7% reduction using biologically synthesised sulfur nanoparticles occurred in 80 min^36^. Another study demonstrated the complete reduction of Cr (VI) in 3 h; however, a considerably low concentration was applied^39^. In the present study, 95.53% reduction of 250 ppm of Cr (VI) was achieved in only 40 min (Fig. 4c).

### Effect of formic acid concentration

**Figure 5:**
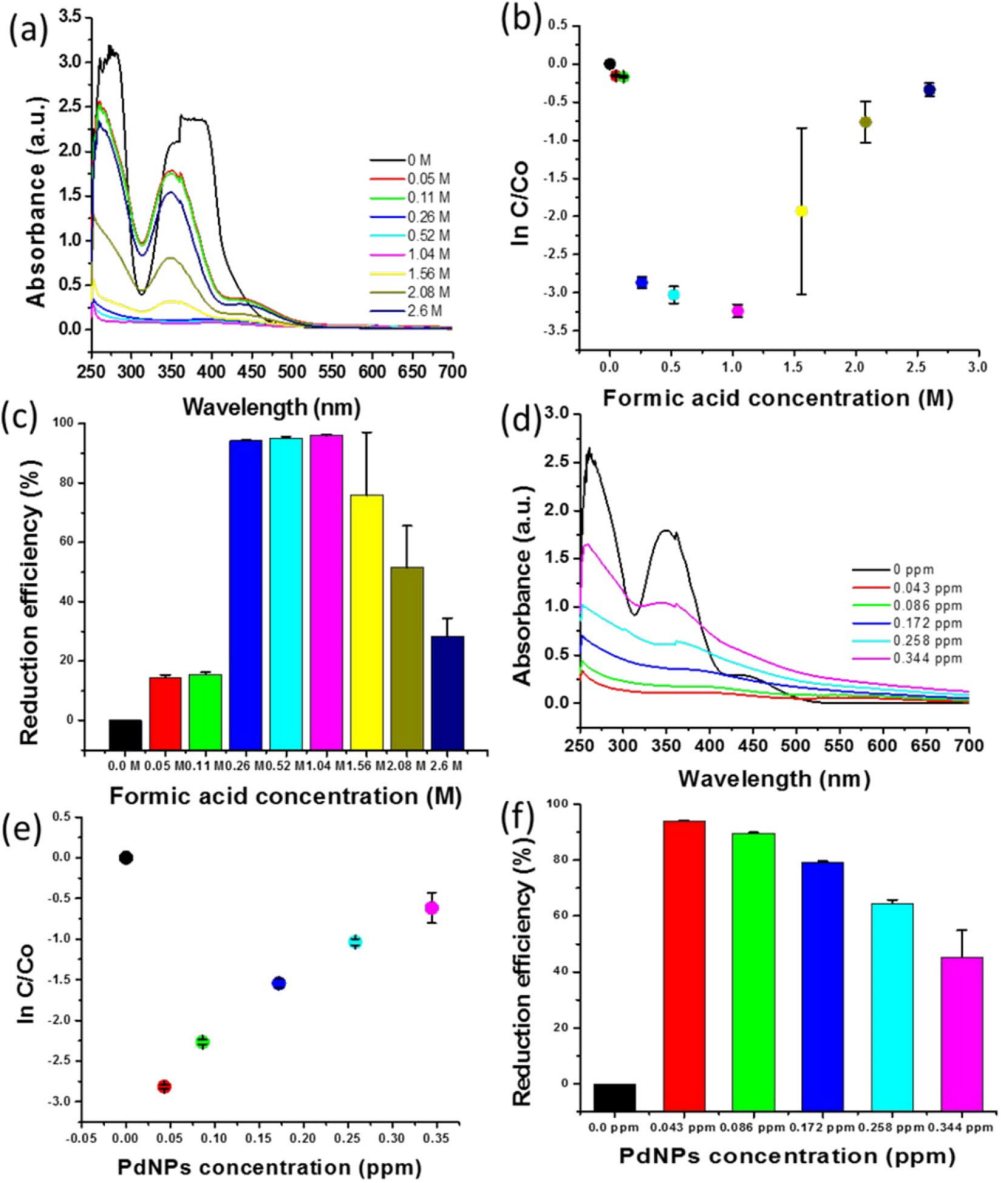
Catalytic reduction efficiency of formic acid and biogenic PdNPs, analysed after 20 min: (**a**) Absorbance spectra changes at 350 nm for Cr (VI) in the presence of various concentrations of formic acid; (**b**) Reduction kinetics, represented by ln(C/C_0_), corresponding to formic acid concentration; (**c**) Reduction efficiency (%) corresponding to formic acid concentration; (**d**) Absorbance spectra changes at 350 nm for Cr (VI) in the presence of various concentrations of PdNPs; (**e**) Reduction kinetics, represented by ln(C/C_0_), corresponding to biogenic-PdNP concentration; (**f**) Reduction efficiency (%) versus PdNP concentration.

We analysed the effect of the formic acid concentration on the catalytic reduction of Cr (VI). Various concentrations of formic acid ranging from 0.05 to 2.6 M were used for the evaluation of the reduction efficiency of Cr (VI) using biogenic PdNPs. The absorption at 350 nm was analysed using the various concentrations of formic acid to determine the optimum concentration of formic acid for the reduction of Cr (VI) (Fig. 5a). Figure 5b shows the effect of the different formic acid concentrations on ln(C/C_0_). The value of ln(C/C_0_) initially decreased with the increase in formic acid concentration; however, above 1.04 M, the value of ln(C/C_0_) increased corresponding to the increase in formic acid concentration, indicating that the reduction efficiency decreased with the increase in formic acid concentration. The greatest decrease in ln(C/C_0_) was observed at formic acid concentrations of 0.26, 0.52, and 1.04 M. The reduction efficiency of Cr (VI) at formic acid concentrations of 0.26, 0.52, and 1.0004 00M demonstrated an approximately similar trend (Fig. 5c). These concentrations exhibited very little difference in reduction efficiency (%); however, the reduction efficiencies at 0.52 and 1.04 M were twice and thrice as high as that at 0.26 M (Fig. 5c); thus, the optimum concentration of formic acid was 0.26 M.

### Effect of Pd nanoparticle concentration

The concentration of the as-synthesised biogenic PdNPs was analysed through inductively coupled plasma-optical emission spectroscopy (ICP-OES). The as-synthesised PdNPs (50 μL) were diluted with 950 μL of deionised water, yielding 0.2152 ppm of PdNPs (Supplementary Table S2). Various concentrations of PdNP, ranging from 0.043 to 0.344 ppm, were used to analyse the effect of the PdNP concentration on the reduction efficiency of Cr (VI). Figure 5d shows the absorption spectra obtained at various concentrations of as-synthesised PdNPs. The lowest absorption was observed at 0.043 ppm, which indicates the highest reduction rate when compared to other concentrations (Fig. 5d). The effect of the PdNP concentration was indicated by the ln(C/C_0_) values corresponding to the as-synthesised PdNPs (Fig. 5e). The reduction efficiency increased with the decrease in ln(C/C_0_). It was observed that the value of ln(C/C_0_) increased for all concentrations of PdNPs, except 0.043 ppm (Fig. 5e). The highest reduction efficiency (%) was observed at 0.043 ppm of the as-synthesised PdNPs (Fig. 5f). Hence, the optimum concentration of PdNPs was determined to be 0.043 ppm.

### Effect of Cr (VI) concentration

**Figure 6:**
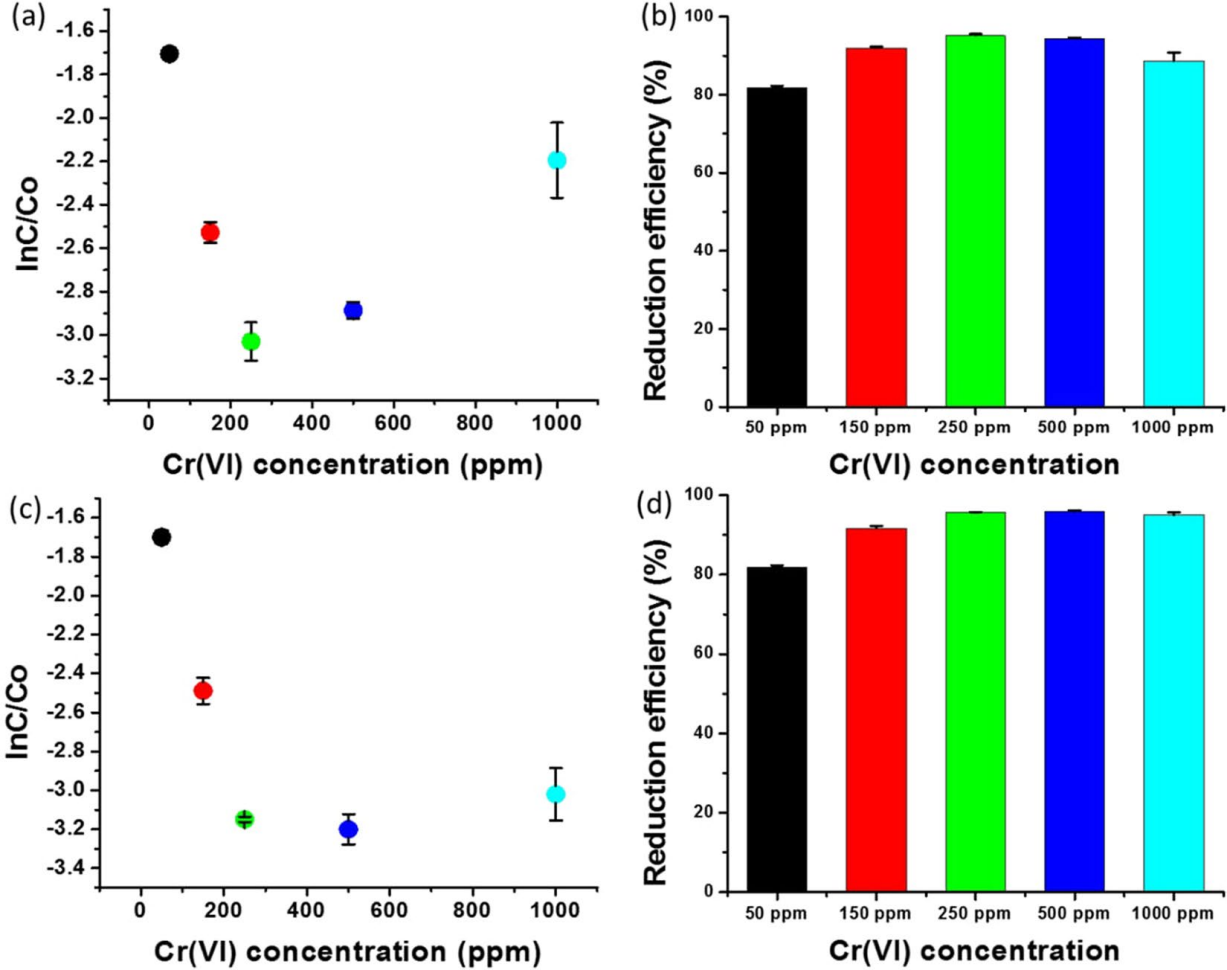
Effect of the Cr (VI) concentration on the reduction efficiency: (**a**) Reduction kinetics, represented by ln(C/C_0_), versus Cr (VI) concentration, analysed after 40 min; (**b**) Reduction efficiency (%) versus Cr (VI) concentration, analysed after 40 min; (**c**) Reduction kinetics, represented by ln(C/C_0_), versus Cr (VI) concentration, analysed after 10 min; (**d**) Reduction efficiency (%) versus Cr (VI) concentration, analysed after 120 min.

The Cr (VI) concentration range of 50–1,000 ppm was used to analyse the reduction efficiency of the combination of 0.043 ppm of PdNPs and 0.26 M of formic acid. The results indicate that the combination of PdNPs and formic acid demonstrated excellent reduction efficiency for a high concentration of Cr (VI). Two sets of experiments were performed with similar concentrations of all the components; however, the sample was analysed at different incubation times: 40 and 120 min. Figure 6a shows that ln(C/C_0_) initially decreased with the increase in Cr (VI) concentration; however, above 250 ppm, ln(C/C_0_) increased with the increase in Cr (VI) concentration. The reduction efficiency was determined to be 95.1% for 250 ppm of Cr(VI), whereas it was 81.2, 92.0, 94.4, and 88.6% for 50, 150, 500, and 1,000 ppm, respectively (Fig. 6b). The second set of samples was analysed after 120 min, which indicated that ln(C/C_0_) decreased with the increase in Cr (VI) concentration (Fig. 6c). The first set exhibited lower reduction efficiency for 500 and 1,000 ppm of Cr (VI) when compared to 250 ppm of Cr (VI), whereas after 120 min, the reduction efficiency was observed to be similar to 250 ppm of Cr (VI) (Fig. 6d). We also analysed the reduction efficiency according to the colorimetric changes. A wide range of Cr (VI) concentrations (12–2,000 ppm) was subjected to reduction with 0.043 ppm of biogenic PdNPs and 0.26 M of formic acid. The yellow colour of the aqueous solution of Cr (VI) disappeared over time (Supplementary Fig. S2). These experimental results indicate that the combination of 0.043 ppm of biogenic PdNPs and 0.26 M of formic acid is excellent for both low and high Cr (VI) concentrations.

### Role of leaf extract

The leaf extract of *E. canadensis* was diluted with deionised water to obtain similar concentrations, which were used for nanoparticles synthesis. The resulting solution was used to analyse the catalytic reduction of Cr (VI) without the addition of PdNPs. The leaf extract was added into an aqueous solution of Cr (VI) in the presence of 0.26 M of formic acid. The absorbance was analysed as a function of time to quantitatively evaluate the reaction kinetics of the leaf extract-mediated catalytic reduction of Cr (VI). The absorbance required up to 40 min, measured at 5 min intervals. Supplementary Fig. S3a shows that the absorption at 350 nm was analogous to the absorption recorded at 0 min. This experiment was performed thrice to obtain error bars for standard deviations, and the data were presented considering the values of ln(C/C_0_) (Supplementary Fig. S3b). The values of ln(C/C_0_) from 0 to 40 min did not show significant differences. Supplementary Fig. S3c shows the reduction efficiency corresponding to time, which clearly indicates a maximum of 0.3% reduction efficiency in 40 min. We also investigated the leaf extract-mediated reduction for 96 h; however, the UV–vis spectra did not show any differences in absorption at 350 nm (Supplementary Fig. S4a). The reduction efficiency without the presence of PdNPs was observed to be only 3% after 96 h, whereas more than 95% reduction was observed after 40 min in the presence of PdNPs (Supplementary Fig. S4b).

### Detection of Cr (III) formation

**Figure 7:**
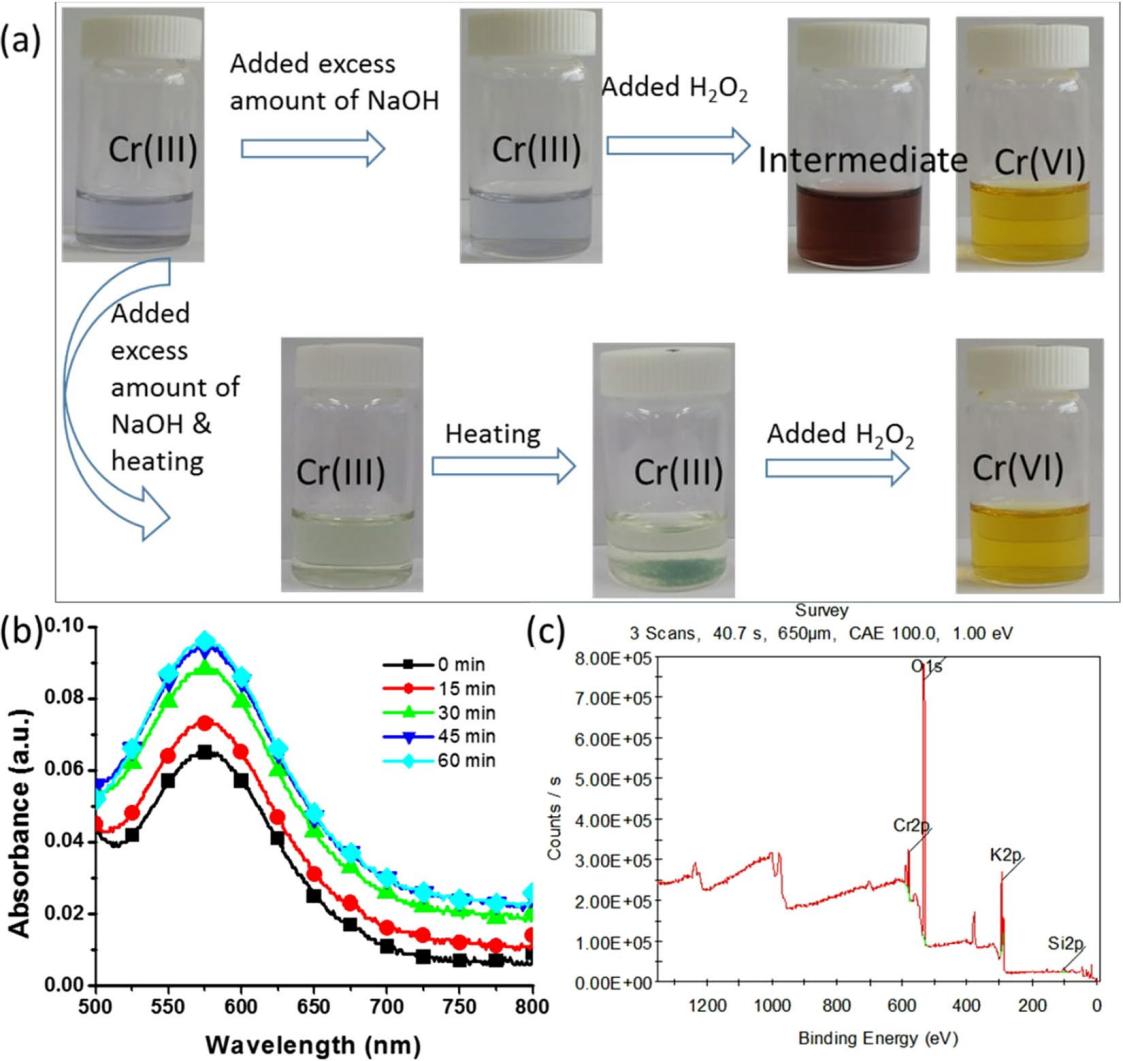
Detection of Cr (III) formation after the catalytic reduction of Cr (VI) using biogenic PdNPs: (**a**) Colorimetric confirmation of Cr (III) formation after the addition of excess amounts of NaOH and H_2_O_2_; (**b**) Increase in the absorbance at 575 nm over time, which is the characteristic peak of Cr (III); (**c**) XPS analysis of the catalytic product.

The formation of Cr (III) by the catalytic reduction of Cr (VI) was confirmed through colorimetric analysis, UV–vis spectroscopy, and XPS analysis of the catalytic product. After the reduction of Cr (VI), the aqueous solution was treated with an excess amount of NaOH solution with and without heating. The aqueous solution of the catalytic product had a light-blue colour, depending on the concentration of Cr (III). When an excess amount of NaOH was added, the colour of the aqueous solution remained the same; however, when H_2_O_2_ was added, the colour of the solution changed to that of wine, and finally turned yellow, which is the characteristic colour of Cr (VI) (Fig. 7a). In the second colorimetric detection of Cr (III), the catalytic product was subjected to a similar treatment with heating. When NaOH was added to the aqueous solution, followed by heating, the solution turned greenish owing to the presence of hexahydroxochromate (III), and further heating caused the precipitation of hexahydroxochromate (III)^44^. With the addition of H_2_O_2_, the green hexahydroxochromate (III) precipitate disappeared, and the solution turned yellow, indicating that the reaction reversed back to Cr (VI) (Fig. 7a). The colorimetric reaction was also investigated using graphs obtained through UV–vis spectroscopy (Supplementary Fig. S5). The formation of Cr (III) was analysed through UV–vis spectroscopy using 500 ppm of Cr (VI) with 0.043 ppm of PdNPs and 0.26 M of formic acid. Figure 7b shows that the absorbance at 575 nm (the characteristic peak of Cr (III)) increased over time, indicating the formation of Cr (III). Furthermore, the catalytic sample was scanned through XPS to determine the oxidation state of Cr. The XPS profiles show a peak at a binding energy of 577.25 eV for the catalytic product, which is the characteristic peak of Cr (III); moreover, no peak corresponding to Cr (VI) was observed (Fig. 7c). These results indicate the formation of Cr (III) by the catalytic reduction of Cr (VI) using the biosynthesised PdNPs. The UV–vis spectra were used to calculate the conversion percentage of Cr (III) and the remaining percentage of Cr (VI). Based on the UV–vis results, we represented the catalytic reduction percentage of Cr species. We applied 250 ppm of Cr (VI) for catalytic reduction and after 40 min, 237.875 ppm was converted into Cr (III), whereas 12.125 ppm remained unchanged in the solution (Supplementary Fig. S6).

### Reusability of catalyst

**Figure 8:**
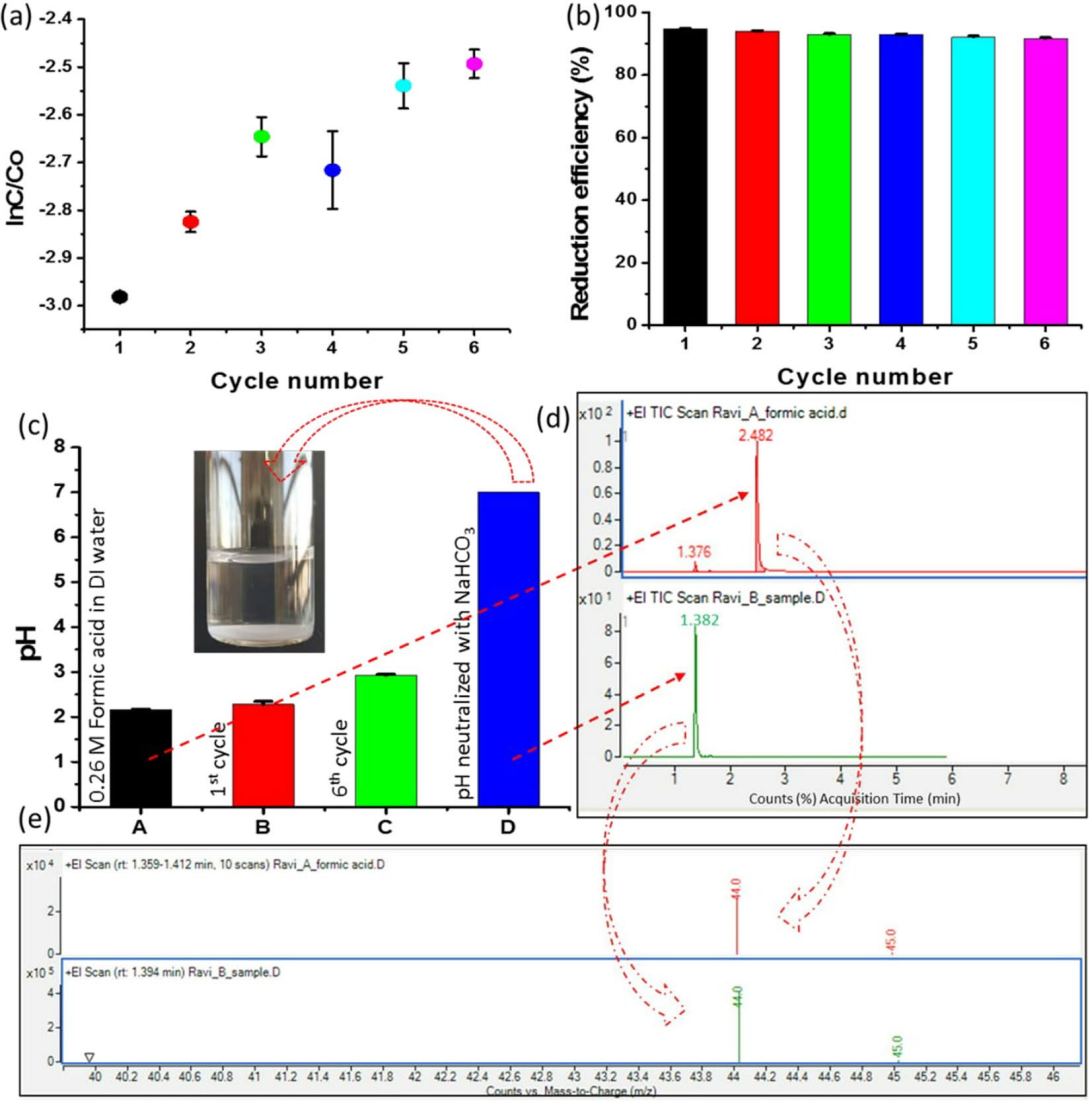
Reusability of the PdNPs without recovery or purification: (**a**) Reduction kinetics represented by ln(C/C_0_) corresponding to the cycle number; (**b**) Reduction efficiency (%) versus the cycle number; (**c**) pH value of solution after the reduction of Cr (VI) (insert shows NaHCO_3_-treated sixth-cycle product); (**d**) Gas chromatography/mass spectrometry analysis for comparison of acquisition time (in minutes) between formic acid and the sixth-cycle solution treated with NaHCO_3_; (**e**) Representation of molecular mass of formic acid and the sixth-cycle solution treated with NaHCO_3_.

We reused the PdNPs for five subsequent cycles of the reduction of Cr (VI). As shown in Fig. 8a, ln(C/C_0_) decreased in the first cycle; however, it showed increased values in subsequent cycles, indicating that the reduction efficiencies were lower in these cycles when compared to that of the first cycle. However, the average reduction efficiency was > 90%, indicating the effectiveness of the biogenic PdNPs (Fig. 8b). Notably, no additional formic acid was used for the subsequent cycles. The formic acid added in the first cycle was sufficient for the subsequent cycles. The addition of formic acid in the subsequent cycles disrupted the equilibrium between the concentrations of formic acid and PdNPs, diminishing the reduction efficiency (Supplementary Fig. S7). We also analysed the reduction efficiency of PdNPs separated through centrifugation at 20,000 revolutions per minute (rpm) for 30 min (Supplementary Fig. S8). The separated PdNPs were used for three subsequent cycles, and ln(C/C_0_) increased after the first cycle, indicating that the reduction efficiency decreased owing to the loss of nanoparticles during the separation from the aqueous solution (Supplementary Fig. S8a,b). Because the proposed method does not require the recovery, purification, or drying of biogenic PdNPs, it is suitable for the large-scale remediation of Cr (VI).

In Fig. 8b, it can be observed that the reduction efficiency decreases significantly slowly, with a reduction of approximately less than 1%. Therefore, a single addition of 0.26 M of formic acid can catalyse several subsequent reduction cycles. The pH of the first cycle was measured after the catalytic conversion of Cr (III); it was observed to be 2.57, which was similar to that for 0.26 M of formic acid in deionised water. After the sixth cycle, the pH had marginally increased and reached a value of approximately 2.95 (Fig. 8c). After the sixth cycle, we neutralised the pH by adding NaHCO_3_ (1 M) before disposing the solution into the environment. Gas chromatography/mass spectrometry (GC/MS) was performed to analyse the presence of formic acid after the treatment of NaHCO_3_. Figure 8d shows that the acquisition time (in minutes) of formic acid was 2.482 (red colour spectrum), whereas that of the NaHCO_3_-treated sixth-cycle product was not the same (green colour spectrum). In the case of the NaHCO_3_-treated sixth-cycle product, the acquisition time was determined to be 1.382; moreover, formic acid showed a considerably small peak because of the presence of carbon dioxide. The molecular masses of formic acid and carbon dioxide are approximately similar; hence, similar peaks were observed for both formic acid and the NaHCO_3_-treated sixth-cycle product (Fig. 8e). It is well known that the reduction of Cr (VI) involves two steps: first is the catalytic hydrogenation of formic acid (HCOOH → H_2_ + CO_2_) and second is the reduction of Cr (VI) to Cr (III) through a H_2_ transfer pathway on the surface of PdNPs. Therefore, GC/MS showed the presence of carbon dioxide in the treated sixth-cycle product.

## Discussion

*E. canadensis* leaf extract was used as a reducing and stabilising agent for the synthesis of PdNPs. As soon as the leaf extract was added to the aqueous solution of palladium chloride at 65 °C under continuous stirring, the colour began to change from yellow to brown. After 30 min, the solution had turned dark brown owing to the excitation of surface plasmon resonance of the PdNPs (Fig. 1a). This shows that the biosynthesis time was 30 min, whereas in a previous study involving the biosynthesis of PdNPs, the peak corresponding to Pd^2+^ ions only vanished after 3 h^45^. Thus, the proposed method allows the rapid biosynthesis of PdNPs.

FTIR analysis was performed to determine the involvement of the biomolecules of the *E. canadensis* leaf extract on the formation of PdNPs. The spectra indicate –OH stretching, C-H stretching of CH_2_, CH_3,_ C = O stretching of the aldehyde group, and COO^−^ stretching vibration (Fig. 1b). This clearly indicate the presence of flavanones adsorbed on the surface of the nanoparticles. The FTIR spectra of the leaf extract and PdNPs revealed that the biological molecules in the leaf extract actively participated in the synthesis of PdNPs.

The TEM images show that all the particles were nanosized and exhibited no agglomeration/aggregation. The biosynthesised nanoparticles had a narrow size distribution from 3 to 25 nm (Fig. 2a) with hexagonal, triangular, and spherical morphologies (Fig. 2b). Figure 2c shows the lattice-fringe characteristic of the crystalline materials, when analysed by HR-TEM. Moreover, the polycrystalline nature of the nanoparticles was observed with 2.27 Å of inter-atomic spacing (d-spacing) of the PdNPs (Fig. 2d, inset on the right). The EDX spectrum confirmed the purity of the biosynthesised PdNPs, as a strong signal of elemental Pd was observed (Fig. 3c). The XPS profiles illustrated that the synthesised nanoparticles were in the zero-valent oxidation state (Fig. 3b).

The biosynthesised PdNPs were used for the catalytic reduction of Cr (VI) to Cr (III) in the presence of formic acid. Conversely, previous studies involved methods to maintain the pH of the solution^32,36,39^, which resulted in difficulty while applying these methods for large-scale wastewater treatment. Table 1 lists the comparative information of previous studies on the catalytic reduction of Cr (VI)^31–39^. In the proposed method, a 250 ppm Cr (VI) aqueous solution was used for catalytic reduction, which is the highest concentration among the studies listed in Table 1. The reduction efficiency was determined to be 95.53% after 40 min of incubation time (Fig. 4c). Supplementary Fig. S1 shows the catalytic activity analysed up to 80 min; however, after 40 min, only a minimal reduction was observed. Therefore, the optimum incubation time was observed to be 40 min for the reduction of 250 ppm of Cr (VI). In a previous study on Cr (VI) reduction using biologically synthesised sulfur nanoparticles, 88.7% reduction was achieved in 80 min^36^. In another study on Cr (VI) reduction, complete reduction was achieved in 3 h with a low concentration of Cr (VI)^39^. In the present study, 95.53% reduction of 250 ppm Cr(VI) was achieved in only 40 min. Therefore, the biologically synthesised PdNPs are promising catalysts for the remediation of Cr contamination.

The effects of formic acid, nanoparticles, and Cr (VI) concentration on catalytic activity were evaluated. Numerous concentrations (0.05–2.6 M) of formic acid were applied to determine the highest reduction efficiency of Cr (VI). The highest decrease in ln(C/C_0_) was observed at formic acid concentrations of 0.26, 0.52, and 1.04 M (Fig. 5b). Therefore, the optimum concentration of formic acid was determined to be 0.26 M, as 0.52 and 1.04 M demonstrated reduction efficiencies that were twice and thrice higher, respectively, at the same ln(C/C_0_). Various concentrations of nanoparticles ranging from 0.043 to 0.344 ppm were used to analyse the reduction efficiency; moreover, the highest reduction efficiency was observed at 0.043 ppm (Fig. 5f). The as-synthesised PdNPs at 0.043 ppm exhibited excellent catalytic reduction of Cr (VI) in the presence of 0.26 M of formic acid. Subsequently, we evaluated the effectiveness of the combination of PdNPs and formic acid for the reduction of various concentrations of Cr (VI). The effect of Cr (VI) concentrations (50–1,000 ppm) on the reduction efficiency was analysed with the combination of 0.043 ppm of PdNPs and 0.26 M of formic acid. We observed that 0.043 ppm of PdNPs and 0.26 M of formic acid act on all the concentrations of Cr (VI); however, the highest reduction was observed for 250 ppm. The reduction efficiencies for 50, 150, 500, and 1,000 ppm, after 40 min, were 81.2, 92.0, 94.4, and 88.6%, respectively (Fig. 6b). The reduction efficiencies of these concentrations were also analysed after 120 min, and were found to be similar to that for 250 ppm of Cr (VI). We observed that if the concentrations of formic acid or PdNPs were increased or decreased for a fixed concentration of 250 ppm of Cr (VI), the reduction efficiency was affected. This implies that PdNPs and formic acid at specific concentrations demonstrated the best results; any increase or decrease in the concentrations acted as an inhibitor for the catalytic reduction of Cr (VI). We determined that 0.043 ppm of PdNPs and 0.26 M of formic acid are the optimum concentrations that work on all concentrations of Cr (VI).

The role of the leaf extract in the reduction of Cr (VI) was evaluated without the presence of PdNPs. A reduction efficiency of only 3% after 96 h was observed without the presence of PdNPs; conversely, more than 95% reduction was observed after 40 min in the presence of PdNPs (Supplementary Fig. S3). Therefore, we concluded that the leaf extract did not influence the reduction of Cr (VI).

Colorimetric detection, UV–vis spectroscopy, and XPS were used to analyse the reduction of Cr (VI) into Cr (III). NaOH was added into the aqueous solution (resultant solution of the Cr (VI) reduction process), followed by heating; the colour of the solution turned greenish because of hexahydroxochromate (III); additionally, heating caused the precipitation of hexahydroxochromate (III)^44^. Further, colorimetric detection was performed by the addition of H_2_O_2_ into the above solution, which caused the green hexahydroxochromate (III) precipitate to disappear; further, the solution turned yellow, representing that the reaction reversed back to Cr (VI) (Fig. 7a). The UV–vis spectra of the resultant solution from Cr (VI) reduction demonstrated a characteristic peak of Cr (III) at 575 nm, with increasing absorbance values over time, indicating the formation of Cr(III).

Reusability is an important property of a catalyst because it indicates the ability to undergo numerous reactions with superior reduction efficiency. Generally, catalysts are separated from the aqueous solution through centrifugation after the completion of a cycle^36^. However, this technique demonstrates disadvantages for large-scale Cr remediation. Therefore, in the present study, the reusability of the catalyst without separation from the solution was examined, as the catalyst was used in an ultralow concentration. The biogenic PdNPs were used in an ultralow amount when compared to previously reported catalysts for Cr remediation (Table 1). Despite the ultralow amount (0.043 ppm), >95% reduction efficiency was achieved. Five subsequent cycles were catalysed by PdNPs for the reduction of Cr (VI), which achieved 90% average reduction efficiency, demonstrating the effectiveness of the biogenic PdNPs (Fig. 8b). We also analysed the reusability by separation and purification of PdNPs; however, satisfactory results were not obtained when compared to the process of purification of nanoparticles without separation. Specifically, no additional formic acid was used for the subsequent cycles as the formic acid added in the first cycle was sufficient for the subsequent cycles.

## Materials and Methods

### Materials

Palladium chloride was purchased from Sigma-Aldrich (USA) and used as a precursor for the synthesis of PdNPs. All other chemicals were analytical-grade and used as received, without purification. Deionised water was used in all the experiments related to the biosynthesis of nanoparticles and catalytic reduction of Cr (VI).

### Leaf-extract preparation

Fresh leaves of *E. canadensis* were chopped (8.5 gm) and dispersed into 100 mL of deionised water in a 200 mL Erlenmeyer flask. Then, the flask was placed on a magnetic stirrer at 500 rpm, and the dispersion was boiled for 40 min. Subsequently, the aqueous mixture solution was allowed to cool at room temperature. Then, the solution was filtered using the Whatman filter paper, yielding the required leaf extract. The leaf extract was stored at 4 °C for utilisation in the biosynthesis of PdNPs.

### Biosynthesis of PdNPs

The glass wares were cleaned to remove the potential nucleation sites using aqua regia solution (1:3 ratio of nitric acid and hydrochloric acid, respectively). Palladium chloride (2.5 mM) was dissolved in 2 mL of ethanol (absolute) using a mixer at 20 rpm for 3 h. The resulting solution was added to 18 mL of deionised water in a 100 mL Erlenmeyer flask, followed by 1 h of stirring (600 rpm) at room temperature. Then, the temperature of the magnetic stirrer was adjusted to 65 °C, and the solution was stirred for 20 min at 400 rpm. Subsequently, 1 mL of the aqueous solution of the leaf extract was added dropwise, followed by stirring under the same conditions for 2 h.

### Characterisation of PdNPs

The biosynthesis of the PdNPs was confirmed by UV–vis spectroscopy (UH5300, Hitachi, Japan) in the scanning range of 300–800 nm. The role of the biological molecules in the synthesis of the PdNPs was examined through FTIR spectroscopy (FTS 7000, Varian, Australia). The size and shape of the PdNPs were characterised through TEM (JEM-3010, JEOL, Japan). The biosynthesised PdNPs were drop-coated on a carbon-coated copper grid for TEM analysis. The elemental composition of the nanoparticles was determined through EDX. The concentration of nanoparticles in the colloidal solution was determined through ICP-OES (Agilent Technologies, USA). XPS analysis (Thermo Scientific, UK) was performed to analyse the oxidation state of the PdNPs.

### Catalytic activity

Formic acid was used for the catalytic conversion of Cr (VI) into a nontoxic state in the presence of PdNPs. Experiments were performed to analyse the catalytic conversion of Cr (VI) into Cr (III). The as-synthesised PdNP colloidal solution was directly used for the conversion of Cr into the nontoxic state. The concentration of PdNPs was analysed through ICP-OES. The sample for ICP-OES analysis comprised 50 μL of the as-synthesised nanoparticles diluted with 950 μL of deionised water. An aqueous solution of Cr (VI) was prepared by dissolving potassium chromate in deionised water. The Cr (VI) concentration range of 50–1,000 ppm was used to analyse the catalytic activity of the biosynthesised PdNPs. The effects of the formic acid, PdNPs, and Cr (VI) concentrations on the catalytic activity were evaluated. UV–vis spectroscopy was performed to analyse the catalytic conversion of Cr (VI) into Cr (III).

### Effect of concentrations

Catalytic activity was influenced by various parameters, including the concentrations of various components. The catalytic conversion of Cr (VI) into Cr (III) involved three components: the aqueous solution of Cr (VI), formic acid, and the colloidal solution of PdNPs. Various concentrations of formic acid (0.05–2.2.6 M), Cr (VI) (50–1,000 ppm), and PdNPs (0.043–0.344 ppm) were evaluated to develop an efficient system for the detoxification of Cr(VI).

### Detection of Cr (III)

The catalytic conversion of Cr (VI) into Cr (III) was evaluated by performing a colorimetric detection assay by adding an excess amount of NaOH solution. After the complete catalytic reaction, the yellow solution turned into a clear or light-blue coloured solution (depending on the concentration of Cr). The clear solution reacted with an excess amount of NaOH solution, causing it to become greenish. For confirmation of the conversion of Cr (VI) into Cr (III), a hydrogen peroxide solution was added, making the solution yellow. For further confirmation, XPS was performed to analyse the oxidation state of the Cr.

### Reusability of PdNPs

The reusability of the catalyst after the completion of the first conversion cycle was tested using two methods: the first method did not require recovery, purification, or drying of the PdNPs, and in the second method, PdNPs were recovered through centrifugation. In the first method, Cr (VI) was added after the first cycle, and in the second method, PdNPs were separated from the solution through centrifugation (20,000 rpm for 30 min). The separated nanoparticles were applied for subsequent cycles to detoxify Cr (VI) with the addition of 0.26 M of formic acid, whereas the first method did not require additional PdNPs or formic acid. The catalytic samples were scanned through UV–vis spectroscopy as a function of time to evaluate the reduction kinetics for the reusability of the PdNPs.

## Supplementary information

Supplementary Information.
