# Supplementary material for: Reclamation of hexavalent chromium using catalytic activity of highly recyclable biogenic Pd(0) nanoparticles
Source: Sci Rep. 2020 Jan 20;10:640. doi: 10.1038/s41598-020-57548-z (PMC6970990; doi:10.1038/s41598-020-57548-z)
Supplement: Supplementary file 1 — Supplementary Information. [file 41598_2020_57548_MOESM1_ESM.pdf]

Reclamation of hexavalent chromium using catalytic activity of highly recyclable biogenic Pd(0) nanoparticles

R.M. Tripathi[1,2] and Sang J. Chung[1]*

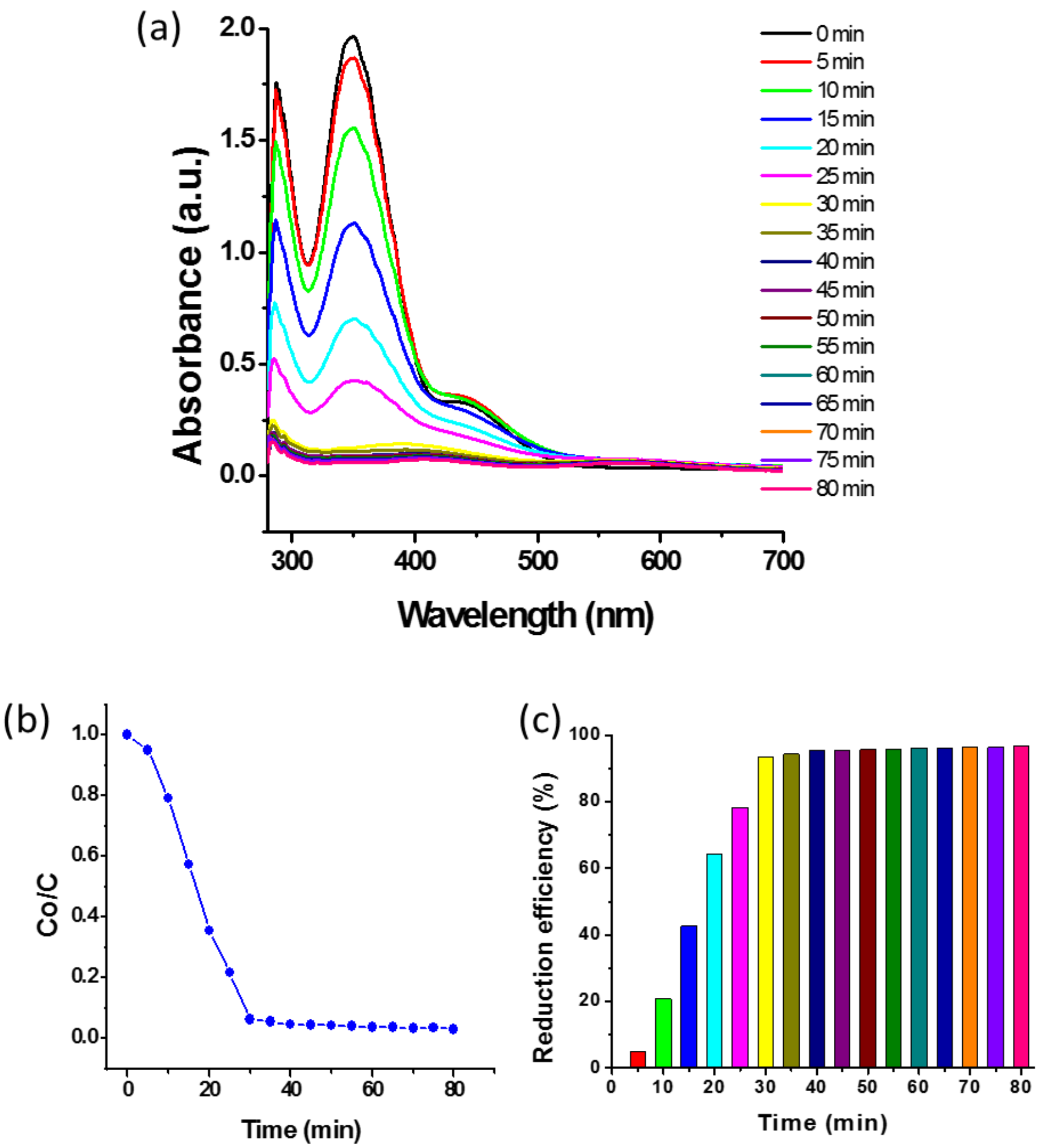

Figure S1. Analysis of the catalytic activity of PdNPs: (**a**) Absorbance spectra changes at 350 nm for Cr (VI) in the presence of PdNPs over time; (**b**) Reduction kinetics of Cr (VI) represented by C/C_0; (**c**) Reduction efficiency (%) versus time

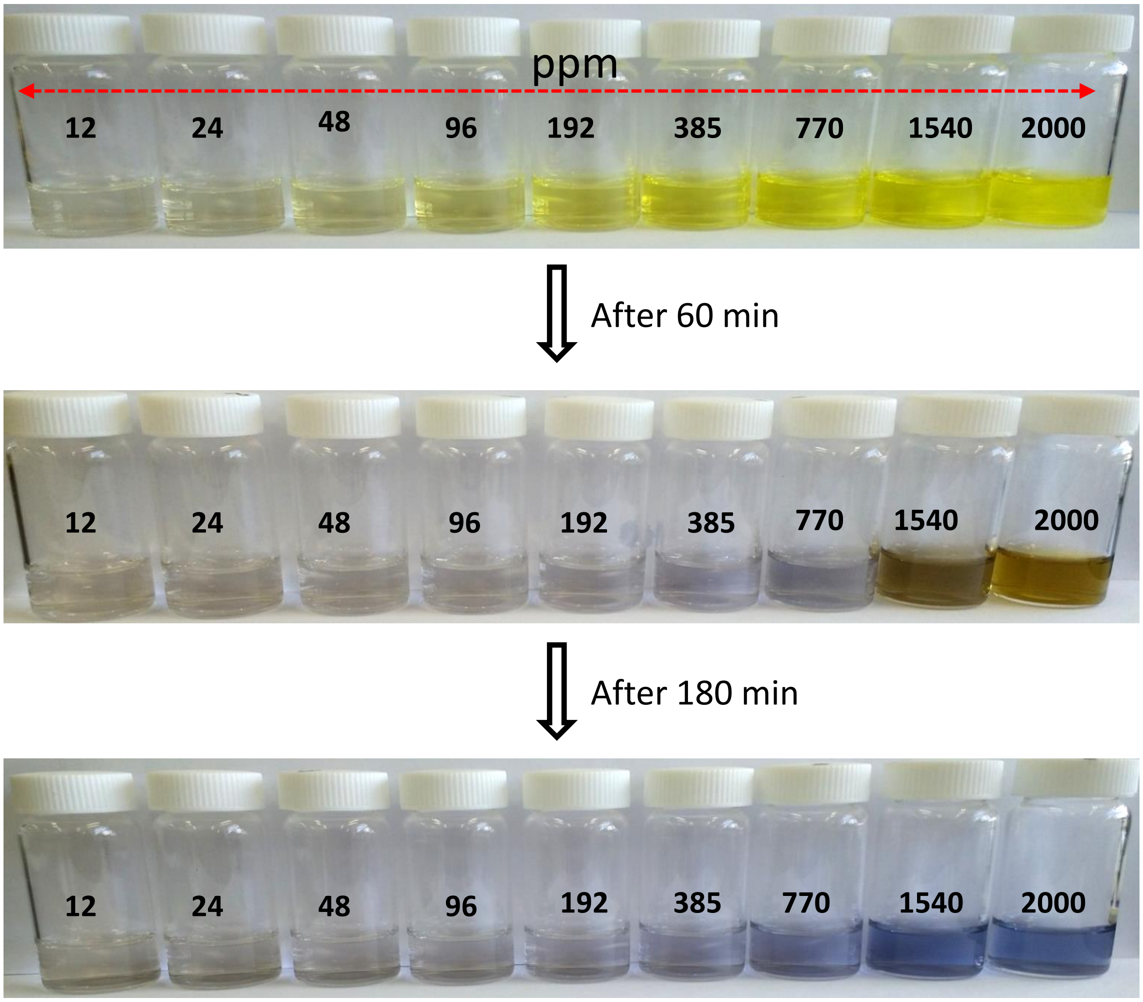

Figure S2. Visible colour changes of various concentrations of Cr (VI) due to the catalytic reduction into Cr (III) using PdNPs (0.043 ppm) in the presence of 0.26 M formic acid

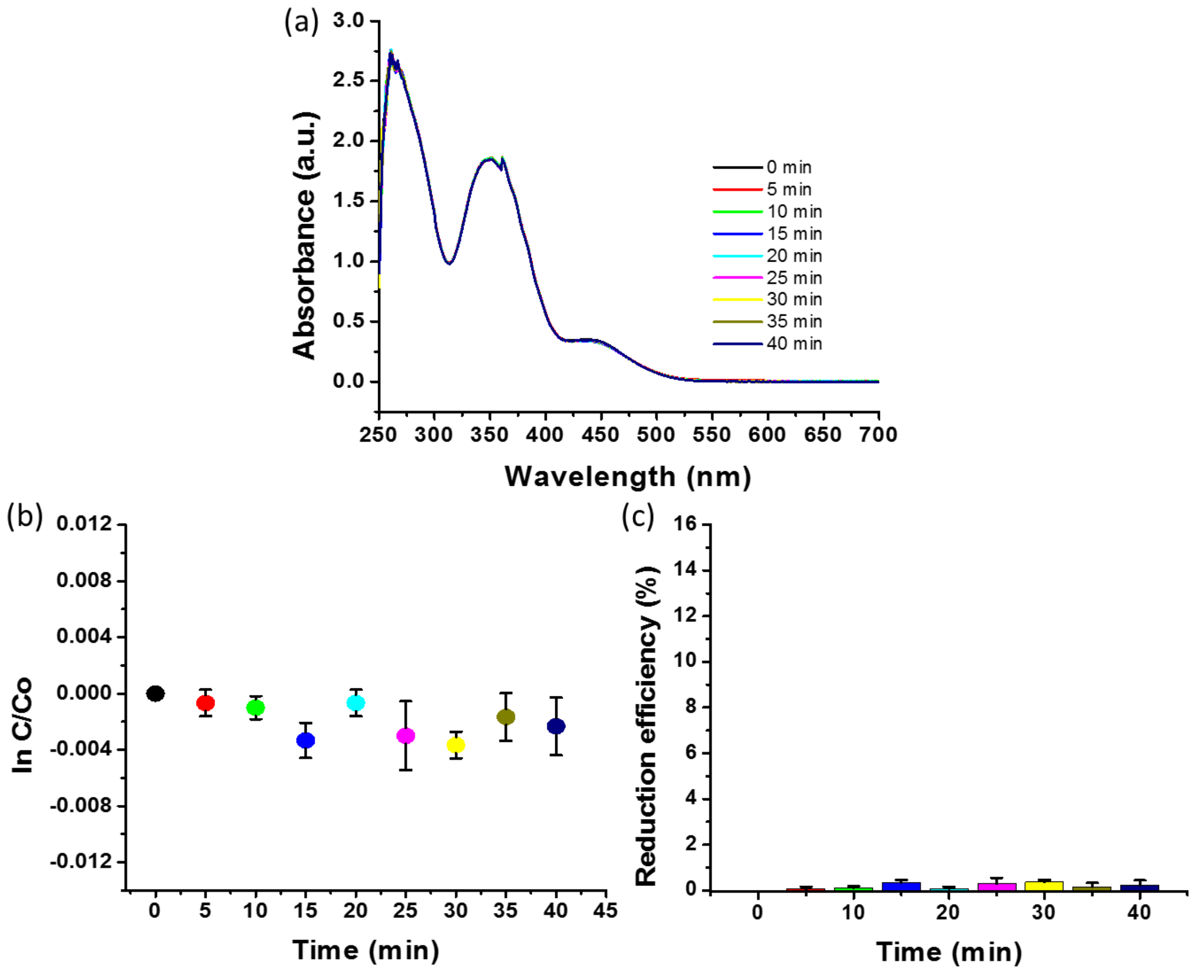

Figure S3. Role of leaf extract in the Cr (VI) reduction after 40 min of incubation: (**a**) Absorbance spectra for Cr (VI) in the presence of leaf extract as a function of time; (**b**) Plot of ln(C/C_0) versus time; (**c**) Reduction efficiency (%) versus time

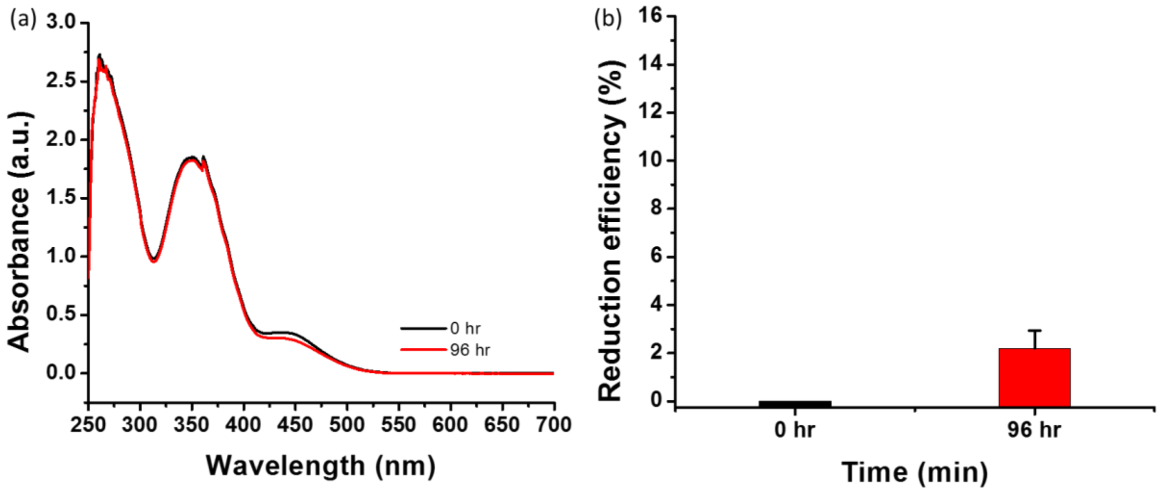

Figure S4. Role of leaf extract in the Cr (VI) reduction after 96 h of incubation: (**a**) absorbance spectra for Cr (VI) in the presence of leaf extract as a function of time; (**b**) reduction efficiency (%) versus time

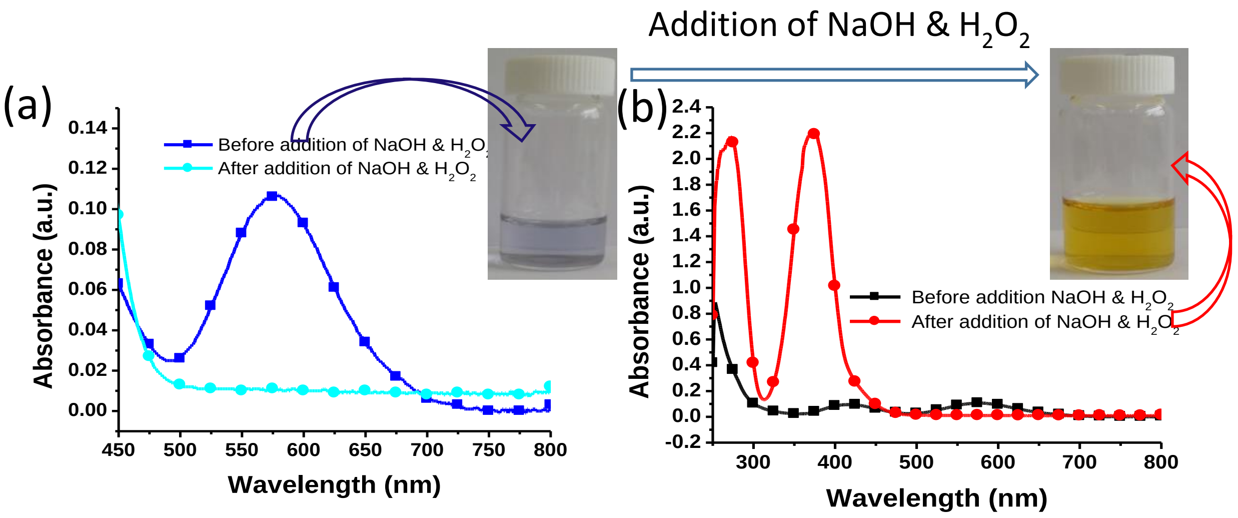

Figure S5. Detection of the catalytic conversion of Cr (VI): (**a**) UV–vis spectra of the catalytic product (inset shows the vial containing the catalytic product); (**b**) UV–vis spectra obtained after the addition of NaOH and H_2O_2 (vial in the inset shows the colour change)

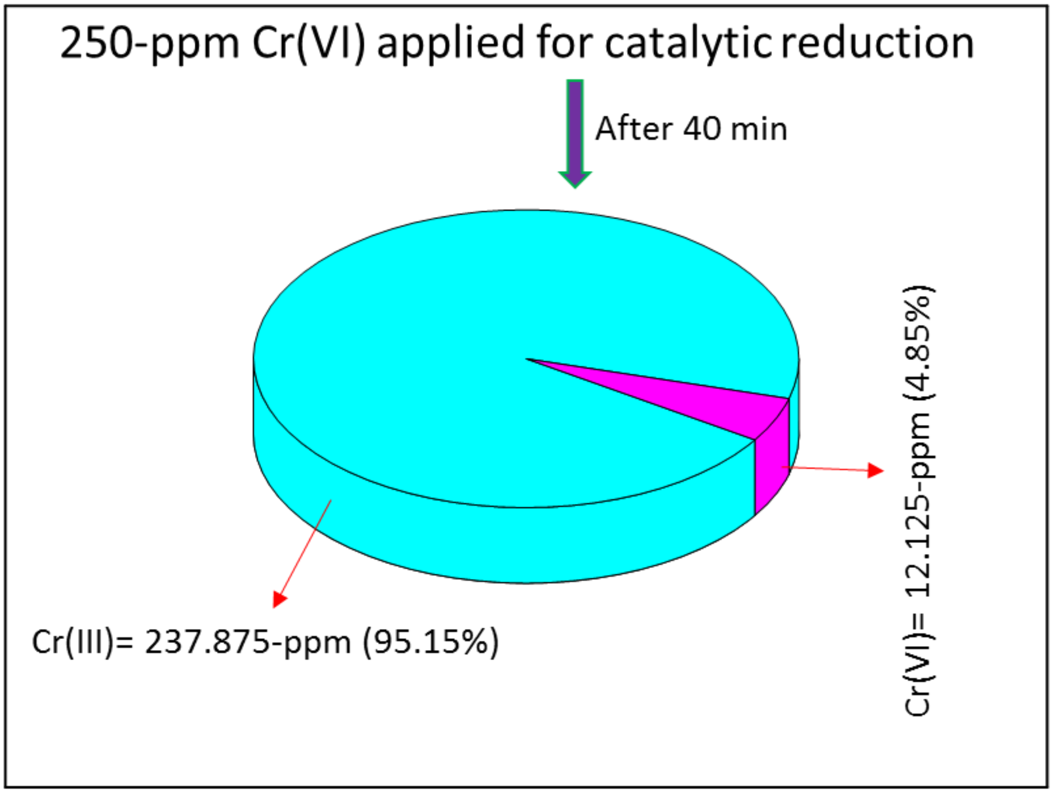

Figure S6. Representation of the catalytic reduction in the form of percentage of Cr species

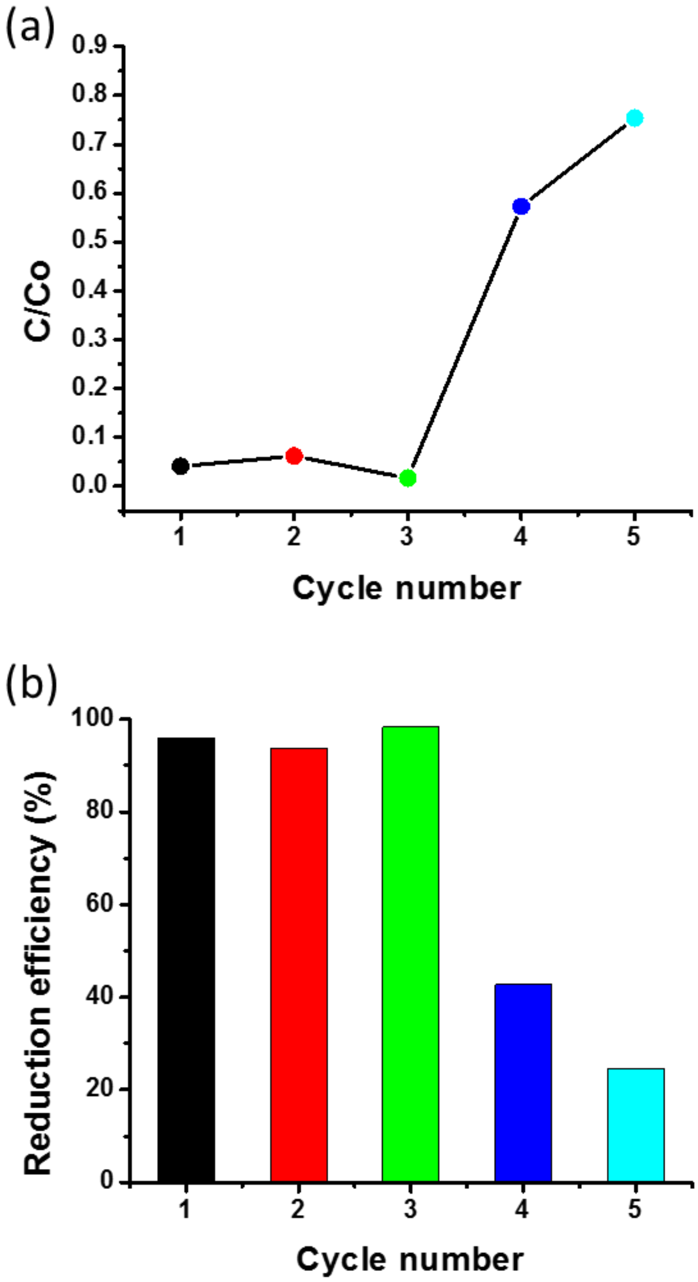

Figure S7. Reusability without recovery or purification, but with the addition of formic acid: (**a**) reduction kinetics represented by C/C_0 versus the cycle number; (**b**) reduction efficiency (%) versus the cycle number

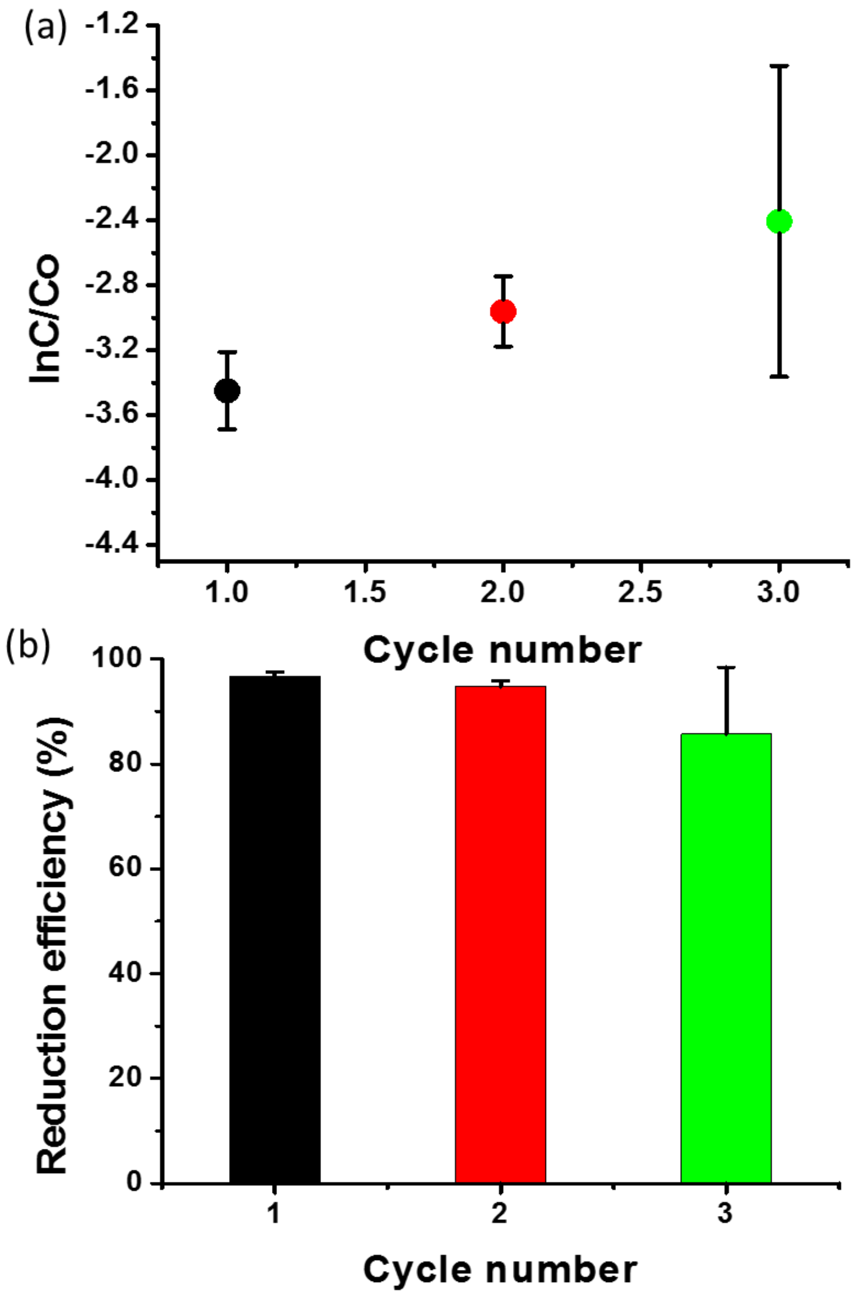

Figure S8. Reusability of the PdNPs after recovery or purification: (**a**) Reduction kinetics represented by ln(C/C_0) versus the cycle number; (**b**) Reduction efficiency (%) versus the cycle number

Element	Line Type	k Factor	Absorption Correction	Wt%	Wt% Sigma	Atomic %
**Cu**	K series	1.233	0.54	14.23	0.43	21.75
**Pd**	L series	1.809	0.62	85.77	0.43	78.25
**Total:**				100.00		100.00

Table S1. Energy-dispersive X-ray analysis results indicating the elemental composition of the biosynthesised Pd nanoparticles (PdNPs)

Label	Element Label	Conc.	%RSD	Unadjusted Conc.	Intensity	%RSD
**Blank**	Pd (340.458 nm)	0.0026 (mg/kg)	12.48	0.0026 (mg/kg)	29.9134	4.84
**Pd**	Pd (340.458 nm)	0.2152 (mg/kg)	1.16	0.2152 (mg/kg)	976.6761	1.14

Table S2. Pd concentration in 1 mL of aqueous solution (50 µL of as-synthesised PdNPs + 950 µL of deionised water)
